# Methyl 1*H*-pyrrole-2-carboxyl­ate

**DOI:** 10.1107/S1600536809030906

**Published:** 2009-08-19

**Authors:** Tobias Kerscher, Peter Mayer, Peter Klüfers

**Affiliations:** a Department Chemie und Biochemie, Ludwig-Maximilians-Universität, Butenandtstrasse 5–13, 81377 München, Germany

## Abstract

The title compound, C_6_H_7_NO_2_, is essentially planar with a dihedral angle of 3.6 (3)° between the pyrrole ring and the methoxy­carbonyl O/C/O/C plane. In the crystal structure, the N atom is a hydrogen-bond donor to the carboxylate C=O O atom of the neighboring mol­ecule. These inter­molecular hydrogen bonds lead to the formation of helical chains along the *b* axis.

## Related literature

For related structures, see: Kerscher, Klüfers, Kügel & Müller (2007 ▶); Kerscher, Klüfers & Kügel (2007 ▶). For graph-set analysis, see: Bernstein *et al.* (1995 ▶); Etter *et al.* (1990 ▶).
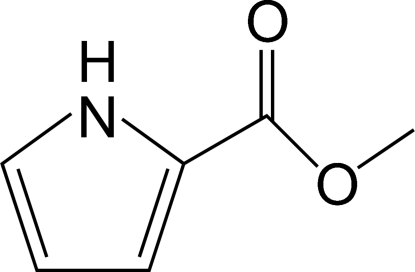


## Experimental

### 

#### Crystal data

C_6_H_7_NO_2_
*M* *_r_* = 125.13
Monoclinic, 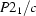

*a* = 7.5346 (19) Å
*b* = 5.4598 (14) Å
*c* = 14.730 (4) Å
β = 100.55 (2)°
*V* = 595.7 (3) Å^3^
*Z* = 4
Mo *K*α radiation
μ = 0.11 mm^−1^
*T* = 200 K
0.38 × 0.16 × 0.06 mm

#### Data collection

Oxford Xcalibur KappaCCD diffractometer
Absorption correction: none
2591 measured reflections
1103 independent reflections
528 reflections with *I* > 2σ(*I*)
*R*_int_ = 0.112

#### Refinement

*R*[*F* ^2^ > 2σ(*F* ^2^)] = 0.070
*wR*(*F* ^2^) = 0.176
*S* = 0.95
1103 reflections
83 parameters
H-atom parameters constrained
Δρ_max_ = 0.27 e Å^−3^
Δρ_min_ = −0.26 e Å^−3^

### 

Data collection: *CrysAlis CCD* (Oxford Diffraction, 2006 ▶); cell refinement: *CrysAlis RED* (Oxford Diffraction, 2006 ▶); data reduction: *CrysAlis RED*; program(s) used to solve structure: *SHELXS97* (Sheldrick, 2008 ▶); program(s) used to refine structure: *SHELXL97* (Sheldrick, 2008 ▶); molecular graphics: *ORTEPIII* (Burnett & Johnson, 1996 ▶), *ORTEP-3* (Farrugia, 1997 ▶) and *Mercury* (Macrae *et al.*, 2006 ▶); software used to prepare material for publication: *SHELXL97*.

## Supplementary Material

Crystal structure: contains datablocks I, global. DOI: 10.1107/S1600536809030906/is2445sup1.cif

Structure factors: contains datablocks I. DOI: 10.1107/S1600536809030906/is2445Isup2.hkl

Additional supplementary materials:  crystallographic information; 3D view; checkCIF report

## Figures and Tables

**Table 1:** Hydrogen-bond geometry (Å, °)

*D*—H⋯*A*	*D*—H	H⋯*A*	*D*⋯*A*	*D*—H⋯*A*
N1—H1⋯O2^i^	0.88	2.06	2.933 (4)	171
C4—H4⋯*Cg*1^ii^	0.95	2.63	3.401 (5)	139

Symmetry codes: (i) 
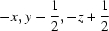
; (ii) 
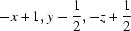
. *Cg*1 is the centroid of the pyrrole ring.

**Fig. 1.**
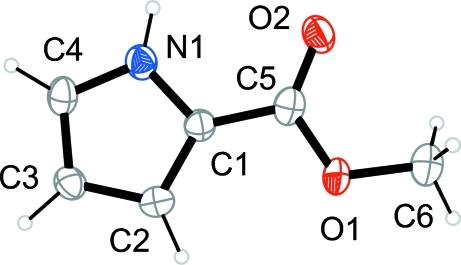
The molecular structure of the title compound with atom labels and anisotropic displacement ellipsoids (drawn at 50% probability level) for non-H atoms.

**Fig. 2.**
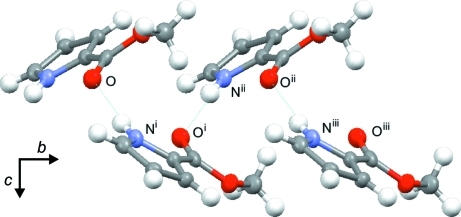
Strands formed by N–H···O hydrogen bonds along [0 1 0] in the crystal structure of the title compound, viewed along [1 0 0]. Symmetry codes: (i) -x, y + 1/2, 1/2 - z; (ii) x, y + 1, z; (iii) -x, y + 3/2, 1/2 - z.

**Fig. 3.**
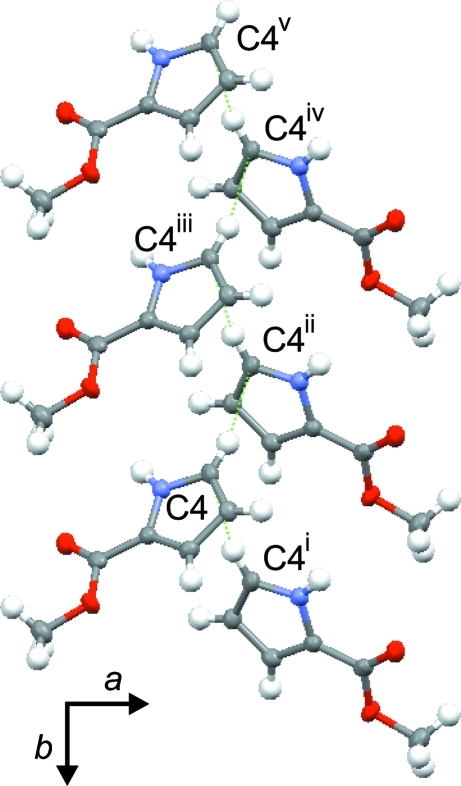
Strands formed by C–H···π contacts along [0 1 0] in the crystal structure of the title compound, viewed along [0 0 1]. Symmetry codes: (i) 1 - x, y + 1/2, 1/2 - z; (ii) 1 - x, y - 1/2, z - 1/2; (iii) x, y - 1, z; (iv) 1 - x, y - 3/2, 1/2 - z; (v) x, y - 2, z.

**Fig. 4.**
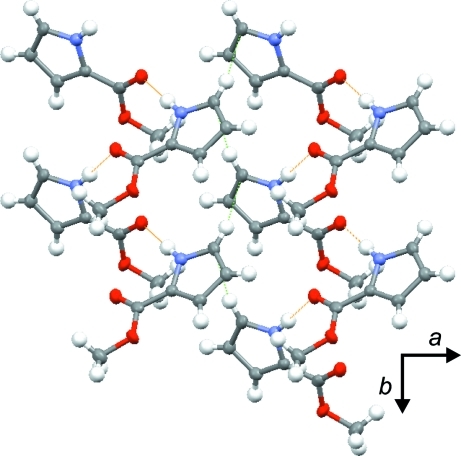
The strands formed by hydrogen bonding in the crystal structure are interconnected by the strands formed by C–H···π contacts.

**Fig. 5.**
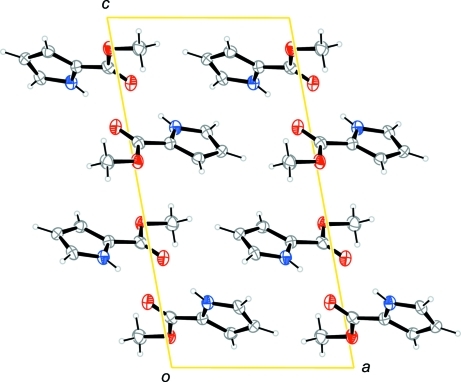
The packing of the title compound, viewed along [0 1 0].

## supplementary crystallographic information

### Comment

The title compound was prepared, in the attempt to create new complexing ligands, as a byproduct. The compound is quite simular to other compounds already published by our group (Kerscher, Klüfers, Kügel & Müller, 2007; Kerscher, Klüfers & Kügel, 2007).

In the molecule, a formic acid methyl ester is in the 2 position of the pyrrole ring (Fig. 1). With a torsion angle for C2–C1–C5–O1 of only about 2.9°, the molecule is, with the exception of the H atoms of the methyl group, nearly planar. Because of this small torsion angle, the molecule is not *C*_s_ symmetric.

The hydrogen bonds of the nitrogen to the carboxylate oxygen lead to a chain like hydrogen bonding system which can be described according to graph-set analysis (Etter *et al.*, 1990; Bernstein *et al.*, 1995) with a C(5) descriptor on the unitary level [the rpluto program (Cambridge Crystallographic Data Centre, England) was used for the graph set analyses; one of these strands is shown in Fig. 2].

Considering contacts whose range falls below the sum of van der Waals radii by only about 0.1 Å, a set of weak C–H···π interactions leads to the formation of a second system of strands along [0 1 0] which can be described by a C(2) descriptor (see Fig. 3).

The two strand systems alternate, which means two hydrogen bonding strands are interconnected by a strand of weak C–H···π contacts (this situation is illustrated in Fig. 4) and *vice versa*, two strands made of weak C–H···π contacts are interconnected by hydrogen bonding strands. This bonding pattern leads to sheet like structures normal to [0 0 1].

The molecular packing of the title compound is shown in Figure 5.

### Experimental

The title compound was obtained by reaction of 228 mg (3.40 mmol) of pyrrole with 280 mg (1.70 mmol) phosgene imminium chloride in 6 ml of dry acetonitrile. After removal of the solvent, the remaining green solid was purified by column chromatoghraphy on silica with chloroform as eluent. Sublimation of fraction five yielded the title compound as colorless crystals.

### Refinement

H atoms were calculated in ideal geometry, with *U*_iso_(H) = 1.2*U*_eq_(C or N) for all aromatic C- and N-bound H atoms, and with *U*_iso_(H) = 1.5*U*_eq_(C) for the methylgroup H atoms.

### Crystal data

**Table:**

C_6_H_7_NO_2_	*F*(000) = 264
*M**_r_* = 125.13	*D*_x_ = 1.395 Mg m^−^^3^
Monoclinic, *P*2_1_/*c*	Mo *K*α radiation, λ = 0.71073 Å
Hall symbol: -P 2ybc	Cell parameters from 1091 reflections
*a* = 7.5346 (19) Å	θ = 4.0–27.5°
*b* = 5.4598 (14) Å	µ = 0.11 mm^−^^1^
*c* = 14.730 (4) Å	*T* = 200 K
β = 100.55 (2)°	Platelet, colourless
*V* = 595.7 (3) Å^3^	0.38 × 0.16 × 0.06 mm
*Z* = 4	

### Data collection

**Table:**

Oxford Xcalibur KappaCCD diffractometer	528 reflections with *I* > 2σ(*I*)
Radiation source: fine-focus sealed tube	*R*_int_ = 0.112
graphite	θ_max_ = 25.5°, θ_min_ = 4.0°
ω–scans	*h* = −9→9
2591 measured reflections	*k* = −6→6
1103 independent reflections	*l* = −17→17

### Refinement

**Table:**

Refinement on *F*^2^	Primary atom site location: structure-invariant direct methods
Least-squares matrix: full	Secondary atom site location: difference Fourier map
*R*[*F*^2^ > 2σ(*F*^2^)] = 0.070	Hydrogen site location: inferred from neighbouring sites
*wR*(*F*^2^) = 0.176	H-atom parameters constrained
*S* = 0.95	*w* = 1/[σ^2^(*F*_o_^2^) + (0.0745*P*)^2^] where *P* = (*F*_o_^2^ + 2*F*_c_^2^)/3
1103 reflections	(Δ/σ)_max_ < 0.001
83 parameters	Δρ_max_ = 0.27 e Å^−^^3^
0 restraints	Δρ_min_ = −0.26 e Å^−^^3^

### Fractional atomic coordinates and isotropic or equivalent isotropic displacement parameters (Å^2^)

**Table:**

	*x*	*y*	*z*	*U*_iso_*/*U*_eq_	
O1	0.0203 (3)	0.7534 (4)	0.08955 (19)	0.0312 (8)	
O2	−0.0644 (3)	0.4755 (5)	0.18723 (19)	0.0331 (8)	
N1	0.2637 (4)	0.2221 (6)	0.1856 (2)	0.0286 (9)	
H1	0.1960	0.1417	0.2183	0.034*	
C1	0.2176 (5)	0.4370 (7)	0.1405 (3)	0.0248 (10)	
C5	0.0452 (5)	0.5519 (7)	0.1425 (3)	0.0276 (10)	
C6	−0.1459 (5)	0.8847 (7)	0.0877 (3)	0.0378 (12)	
H6A	−0.2475	0.7701	0.0753	0.057*	
H6B	−0.1594	1.0093	0.0391	0.057*	
H6C	−0.1439	0.9640	0.1476	0.057*	
C3	0.4923 (5)	0.3236 (7)	0.1182 (3)	0.0275 (10)	
H3	0.6054	0.3212	0.0984	0.033*	
C4	0.4295 (5)	0.1506 (7)	0.1726 (3)	0.0300 (10)	
H4	0.4914	0.0065	0.1967	0.036*	
C2	0.3585 (5)	0.5042 (7)	0.0975 (3)	0.0276 (10)	
H2	0.3638	0.6461	0.0608	0.033*	

### Atomic displacement parameters (Å^2^)

**Table:**

	*U*^11^	*U*^22^	*U*^33^	*U*^12^	*U*^13^	*U*^23^
O1	0.0235 (16)	0.0309 (17)	0.0388 (19)	0.0084 (12)	0.0042 (12)	0.0051 (13)
O2	0.0241 (16)	0.0352 (17)	0.041 (2)	0.0013 (13)	0.0090 (14)	−0.0012 (14)
N1	0.025 (2)	0.0251 (19)	0.036 (2)	−0.0002 (15)	0.0059 (15)	−0.0020 (16)
C1	0.024 (2)	0.022 (2)	0.028 (2)	−0.0012 (18)	0.0041 (18)	−0.0015 (17)
C5	0.028 (2)	0.022 (2)	0.030 (2)	0.0005 (18)	−0.0009 (19)	−0.0072 (18)
C6	0.034 (3)	0.037 (3)	0.041 (3)	0.009 (2)	0.004 (2)	0.001 (2)
C3	0.026 (2)	0.031 (2)	0.026 (2)	0.0016 (18)	0.0079 (17)	−0.0016 (19)
C4	0.023 (2)	0.029 (2)	0.035 (3)	0.0024 (19)	−0.0013 (18)	−0.0029 (19)
C2	0.030 (2)	0.026 (2)	0.026 (3)	−0.0026 (19)	0.0051 (19)	0.0019 (19)

### Geometric parameters (Å, °)

**Table:**

O1—C5	1.341 (4)	C6—H6A	0.9800
O1—C6	1.439 (4)	C6—H6B	0.9800
O2—C5	1.221 (5)	C6—H6C	0.9800
N1—C4	1.356 (5)	C3—C4	1.377 (5)
N1—C1	1.362 (5)	C3—C2	1.403 (5)
N1—H1	0.8800	C3—H3	0.9500
C1—C2	1.382 (5)	C4—H4	0.9500
C1—C5	1.447 (5)	C2—H2	0.9500
			
C5—O1—C6	116.6 (3)	O1—C6—H6C	109.5
C4—N1—C1	109.8 (3)	H6A—C6—H6C	109.5
C4—N1—H1	125.1	H6B—C6—H6C	109.5
C1—N1—H1	125.1	C4—C3—C2	107.4 (3)
N1—C1—C2	107.7 (3)	C4—C3—H3	126.3
N1—C1—C5	120.9 (3)	C2—C3—H3	126.3
C2—C1—C5	131.4 (4)	N1—C4—C3	108.0 (3)
O2—C5—O1	123.9 (4)	N1—C4—H4	126.0
O2—C5—C1	124.1 (4)	C3—C4—H4	126.0
O1—C5—C1	112.0 (4)	C1—C2—C3	107.2 (3)
O1—C6—H6A	109.5	C1—C2—H2	126.4
O1—C6—H6B	109.5	C3—C2—H2	126.4
H6A—C6—H6B	109.5		
			
C4—N1—C1—C2	0.0 (4)	C2—C1—C5—O1	2.9 (6)
C4—N1—C1—C5	179.3 (3)	C1—N1—C4—C3	0.3 (4)
C6—O1—C5—O2	0.7 (5)	C2—C3—C4—N1	−0.4 (4)
C6—O1—C5—C1	−179.0 (3)	N1—C1—C2—C3	−0.2 (4)
N1—C1—C5—O2	4.1 (6)	C5—C1—C2—C3	−179.4 (4)
C2—C1—C5—O2	−176.8 (4)	C4—C3—C2—C1	0.4 (4)
N1—C1—C5—O1	−176.2 (3)		

### Hydrogen-bond geometry (Å, °)

**Table:**

*D*—H···*A*	*D*—H	H···*A*	*D*···*A*	*D*—H···*A*
N1—H1···O2^i^	0.88	2.06	2.933 (4)	171
C4—H4···Cg1^ii^	0.95	2.63	3.401 (5)	139

Symmetry codes: (i) −*x*, *y*−1/2, −*z*+1/2; (ii) −*x*+1, *y*−1/2, −*z*+1/2.
